# The role of YY1 in reduced HP1α gene expression in invasive human breast cancer cells

**DOI:** 10.1186/bcr2329

**Published:** 2009-06-30

**Authors:** Jason G Lieberthal, Marissa Kaminsky, Christopher N Parkhurst, Naoko Tanese

**Affiliations:** 1 Department of Microbiology and NYU Cancer Institute, New York University School of Medicine, 550 First Avenue, New York, NY 10016

## Abstract

**Introduction:**

Heterochromatin protein 1 (HP1) associates with chromatin by binding to histone H3 and contributes to gene silencing. There are three isoforms of HP1 in mammals: HP1α, β, and γ. Studies have shown that the level of HP1α is reduced in invasive human breast cancer cell lines such as MDA-MB-231 and HS578T compared with non-invasive cell lines such as MCF7 and T47D. It is hypothesized that reduced HP1α expression may lead to impaired epigenetic silencing of genes that are important in the acquisition of an invasive phenotype. We set out to determine whether reduced expression of HP1α in invasive breast cancer cell lines occurs at the level of transcription.

**Methods:**

We used transient transfection assays to investigate the mechanism of differential transcriptional activity of the human HP1α gene promoter in different cell lines. Mutational analysis of putative transcription factor binding sites in an HP1α gene reporter construct was performed to identify transcription factors responsible for the differential activity. SiRNA-mediated knockdown and chromatin immunoprecipitation experiments were performed to determine the role of a specific transcription factor in regulating the HP1α gene.

**Results:**

The transcription factor yin yang 1 (YY1) was found to play a role in differential transcriptional activity of the HP1α gene. Examination of the YY1 protein and mRNA levels revealed that both were reduced in the invasive cell line HS578T compared with MCF7 cells. YY1 knockdown in MCF7 cells resulted in a decreased level of HP1α mRNA, indicating that YY1 positively regulates HP1α expression. Chromatin immunoprecipitation experiments verified YY1 occupancy at the HP1α gene promoter in MCF7 cells but not HS578T cells. Overexpression of YY1 in HS578T cells decreased cell migration in a manner independent of HP1α overexpression.

**Conclusions:**

Our data suggests that a reduction of YY1 expression in breast cancer cells could contribute to the acquisition of an invasive phenotype through increased cell migration as well as by reduced expression of HP1α.

## Introduction

Heterochromatin protein 1 (HP1) was first identified in *Drosophila *as a non-histone component of chromatin [1]. Mutations in the HP1 gene resulted in suppression of position-effect variegation, a result that implicated HP1 in chromatin structure and gene expression [2]. Mutation of the gene encoding HP1 in *Drosophila *resulted in larval lethality [3]. Examination of HP1 mutant embryos revealed defects in chromosome segregation and telomere maintenance [4,5]. Therefore, HP1 is thought to play an essential role in heterochromatin-dependent processes in *Drosophila*. HP1 can also be found in certain euchromatic loci, implying its role in euchromatic regions [6,7].

HP1 homologues have been identified in a variety of organisms including yeast, nematodes, insects, chickens, frogs, and mammals [8]. There are three HP1 isoforms in mammals: HP1α, β and γ [9,10]. Each HP1 isoform has a different chromosomal distribution. HP1α is located mainly in heterochromatic regions, HP1β is found in both heterochromatic and euchromatic regions, and HP1γ is located almost exclusively in euchromatic regions [11-13]. The localization of HP1 isoforms to different regions of chromatin implies that each isoform plays a unique role in chromatin structure and transcriptional regulation.

All HP1 family members share a similar structure: an amino-terminal chromodomain (CD), a variable hinge region and a carboxy-terminal chromoshadow domain (CSD) [8]. HP1 associates with chromatin primarily through the CD, which binds to the histone-fold domain of histone H3 [13,14]. This interaction is stimulated by methylation of the H3 histone tail on lysine 9 [15,16]. It has therefore been suggested that the repressive effect of H3K9 methylation is mediated, in part, by HP1. HP1α can also interact with histone H1 [13,17,18]. In addition, RNA may play a role in targeting HP1α to pericentric heterochromatin by interacting with the hinge region [19]. An interaction between HP1α and the histone variant H2A.Z may contribute to the compaction of heterochromatic domains [20]. However, the mechanism by which different isoforms of HP1 occupy distinct regions of chromatin remains unclear.

Although HP1 associates with chromatin via the CD, the CSD of HP1 can mediate interactions with a number of different proteins [21]. The CSD can bind HP1 itself, allowing HP1 to hetero- and homo-dimerize [13]. This interaction is thought to contribute to the compaction of heterochromatic domains. The CSD can also bind the histone methyltransferase SUV39H1, an interaction that may facilitate spreading of heterochromatin to adjacent loci [22,23]. The CSD mediates the interaction between HP1 and the co-repressor KAP-1 (TIF1β, KRIP1), which can result in mitotically-heritable gene silencing [24,25]. Interaction between HP1α and γ and the TFIID subunit TAF4 (TAF_II_130) is also mediated by the CSD and may be responsible for dissociation of TAF4 from promoter regions upon HP1 binding [26,27]. The ability of the CSD to associate with such a functionally diverse group of proteins suggests that HP1 exerts its effects on gene expression through a variety of mechanisms.

It has been reported that HP1α expression is reduced in invasive human breast cancer cell lines such as HS578T and MDA-MB-231 compared with non-invasive breast cancer cell lines such as MCF7 and T47D [28]. Over-expression of HP1α in the invasive cell line MDA-MB-231 reduced its *in vitro *invasive potential [29]. Reducing the expression of HP1α in MCF7 cells increased their invasive potential without affecting their rate of growth [30]. This data suggests that HP1α acts as a metastasis suppressor in breast cancer cells. In addition, reduction of HP1α expression has been observed in metastatic colon cancer cell lines compared with non-metastatic cell lines, in desmoplastic vs. classic meduloblastoma, and in papillary thyroid carcinoma compared with normal thyroid tissue [31-33].

HP1α is one of many proteins that have been identified as metastasis suppressors. These proteins have roles in diverse cellular functions including cell adhesion and migration as well as cell signaling [34]. The role of HP1α in epigenetic gene silencing makes it a unique metastasis suppressor. A decrease in HP1α expression could disrupt the epigenetic program of the cell, altering gene expression at a global level. Therefore, it has been hypothesized that decreased expression of HP1α in breast cancer cells leads to deficient epigenetic silencing of genes that promote a metastatic phenotype. We set out to determine the mechanism by which HP1α expression is reduced in highly invasive breast cancer cells.

To address this question we used the MCF7 and HS578T breast cancer cell lines as models of non-invasive and invasive breast cancer, respectively. To study the transcriptional activity of the HP1α gene promoter in these cell lines we sub-cloned the human HP1α gene promoter region into a luciferase reporter construct. Using the Transcription Element Search Software (TESS) database, we identified several highly conserved transcription factor binding motifs in the HP1α promoter region [35]. We then used site-directed mutagenesis to assess the importance of each motif in the transcriptional activity of the HP1α gene promoter in each cell line. Our study suggests that the transcription factor yin yang 1 (YY1) may be involved in the differential expression of HP1α in MCF7 and HS578T cells. In addition, we demonstrate that YY1 overexpression suppresses HS578T cell migration *in vitro*. We conclude that decreased YY1 expression may contribute to the invasive phenotype of metastatic breast cancer cells.

## Materials and methods

### Cell culture

Cell lines were obtained from American Type Culture Collection (ATCC) and maintained in Dulbecco's Modified Eagle Medium (DMEM; Invitrogen, Carlsbad, CA, USA) supplemented with 10% fetal bovine serum (Gemini Bio-Products, West Sacramento, CA, USA) and 1% penicillin/streptomycin/glutamine mixture (Invitrogen, Carlsbad, CA, USA). Culture medium for the HS578T cell line was supplemented with 0.01 mg/ml bovine insulin (Invitrogen, Carlsbad, CA, USA).

### Analysis of the HP1α gene promoter region

The University of California Santa Cruz (UCSC) genome browser [36] was used to determine which nucleotides in the HP1α promoter region are conserved between human, mouse, rat and chimpanzee. The sequence of the HP1α gene promoter region was entered into the TESS database to identify transcription factor binding motifs [35]. Motifs with high similarity to a consensus binding site and high sequence conservation between species were noted.

### Construction of reporter constructs

Portions of the HP1α gene promoter region were PCR-amplified from a BAC clone [GenBank:AC078778] containing a portion of human chromosome 12. The PCR-amplified fragments were sequenced and ligated into the pGL3 luciferase reporter vector (Promega, Madison, WI, USA) digested with HindIII and XhoI. Primer sequences are available upon request.

### Site-directed mutagenesis

The QuikChange Site-Directed Mutagenesis kit (Stratagene, La Jolla, CA, USA) was used according to the manufacturer's instructions. Mutations in transcription factor binding sites were as follows: site YY1.1 was changed from AAATGG to AAGCTT, site YY1.2 was changed from AAAATGGCG to AAAGCTTCG, the E-box site was changed from CACGTG to CGATCG, and site NRF-1.3 was changed from TGCGCAGGCGCA to TGCGCATATGCA. In each case, nucleotides reported to be critical for factor binding were changed. The YY1.2 mutation abolished YY1 binding (see text), and the NRF-1.3 mutation abolished NRF-1 binding as determined by electrophoretic mobility shift assay (EMSA) (data not shown).

### Real time RT-PCR

RT-PCR was performed using the Roche LightCycler instrument. Reactions were carried out in LightCycler capillaries (20 μl) (Roche, Indianapolis, IN, USA) and contained SYBR. Green Taq ReadyMix (Sigma, St. Louis, MO, USA), Enhanced avian reverse transcriptase (Sigma, St. Louis, MO, USA) (2 units/reaction), and 250 nM forward and reverse primers. Reactions contained 400 ng of template RNA with the exception of the 28S-specific reactions, which contained 5 ng of template RNA. Reverse transcription was performed at 61°C followed by a 95°C denaturation step. The annealing temperature for all reactions was 60°C.

### Western blotting

Concentrations of protein samples were quantified using Bradford reagent (BioRad, Hercules, CA, USA). Samples were run on gels containing 8 or 10% acrylamide (National Diagnostics, Atlanta, GA, USA) and then transferred to nitrocellulose membranes, which were blocked with tris-buffered saline with Tween (TBST) containing 5% milk. The membranes were probed with antibodies against HP1α (Millipore #07-346, Billerica, MA, USA), YY1 (Santa Cruz Biotechnology sc-7341X, Santa Cruz, CA, USA), hnRPA1 (Abcam ab5832, Cambridge, MA, USA), or β-tubulin (Covance TU27 MMS410P, Princeton, NJ, USA). Signal was detected using horseradish peroxidase-conjugated secondary antibody and developed using the BioRad Immun-Star reagents (Hercules, CA, USA).

### Luciferase and β-galactosidase assays

Cells were plated in 35 mm culture dishes (10^5 ^cells/dish). On the following day cells were transfected with plasmid DNA (0.5 μg total) using 1.5 μl TransIT-LT1 reagent (Mirus, Madison, WI, USA) per plate. Each plate was transfected with 0.25 μg of luciferase reporter plasmid and 0.25 μg of the CMV-β-galactosidase plasmid. The final concentration of plasmid DNA was 0.5 ng/μl. After 6 to 8 hours cells were washed with phosphate-buffered saline (PBS) and re-fed. The next day plates were washed with PBS and cells were harvested using 0.4 ml Triton/Gly-Gly lysis buffer (25 mM Gly-Gly, pH 7.8; 15 mM MgSO_4_; 4 mM ethylene glycol tetraacetic acid (EGTA); 1% Triton X-100; 1 mM dithiothreitol (DTT)). Luciferase assays were performed by adding 50 μl of cell lysate to 300 μl of luciferase reaction solution (27 mM Gly-Gly, pH 7.8; 16 mM MgSO_4_; 0.1 mg/ml BSA; 1 mM DTT; 1 mM ATP). The sample was put in a luminometer (EG&G Berthold Lumat LB 9507, Oak Ridge, TN, USA), which injected 100 μl of 1 mM D-luciferin into each sample and measured the light emission for 10 seconds. β-galactosidase assays were carried out by adding 50 μl of cell lysate to 500 μl of Lac-Z reaction buffer (100 mM sodium phosphate, pH 6.95; 10 mM KCl; 1 mM MgSO_4_; 0.21% β-mercaptoethanol), followed by addition of 100 μl of o-nitrophenyl-β-D-galactopyranoside (ONPG) (4 mg/ml). The reactions were stopped by addition of 1 M Na_2_CO_3 _and quantified by measuring the absorbance at 420 nm. Transfection and reporter assays were performed at least three times and the result of a representative experiment is shown.

### siRNA knockdown experiments

MCF7 cells were plated in 60 mm dishes (10^5 ^cells/dish). The following day the cells were transfected with siRNA using Oligofectamine reagent (Invitrogen, Carlsbad, CA, USA). siRNA oligos were diluted to 500 nM with Opti-MEM (Invitrogen, Carlsbad, CA, USA). Oligofectamine reagent (2.8 μl/plate) was diluted approximately 1:100 in Opti-MEM and allowed to stand at room temperature for five minutes. siRNA and Opti-MEM dilutions were then mixed at a 1:1 ratio and allowed to stand at room temperature for 20 minutes before adding to plates of cells. The final concentration of siRNA in each plate was 50 nM. The following day the plates were washed with PBS and re-fed with DMEM containing 10% fetal bovine serum. Five days after transfection RNA and protein were isolated using TRI Reagent (Sigma, St. Louis, MO, USA) according to the manufacturer's instructions. siRNA oligos were purchased from Dharmacon and were specific for luciferase (sense strand 5'-CUUACGCUGAGUACUUCGA-3') or YY1 (oligo 59: sense strand 5'-AAGAUGAUGCUCCAAGAAC-3'; oligo OT: sense strand 5'-CAUAAAGGCUGCACAAAGA-3').

### Electrophoretic mobility shift assay

Probes were prepared by end-labeling each oligo (wild type probe: 5'-GCGCAAAACTCGCCATTTTACTACACG-3' and its complementary sequence; YY1 mutant probe: 5'-GCGCAAAACTCGAAGCTTTACTACACG-3' and its complementary sequence) with γ-^32^P ATP using T4 polynucleotide kinase (Promega, Madison, WI, USA). Radiolabeled probes were purified using Quick Spin Columns (Roche, Indianapolis, IN, USA). A 6% TBE (tris, boric acid, edta)-polyacrylamide gel was poured and allowed to polymerize overnight. Nuclear extract (25 μg) was incubated on ice for 15 minutes in EMSA buffer (6 mM Tris, pH 8.0; 6 mM MgCl_2_; 150 mM NaCl; 1 mM DTT) containing 10 μg/ml BSA, 10 μg/ml poly (dI-dC), 50 μg/ml salmon sperm DNA (Invitrogen, Carlsbad, CA, USA). One μg of antibody against AcK9H3 (Upstate #06-599) or YY1 (Santa Cruz sc-7341X) was then added to the appropriate samples, and all samples were incubated at room temperature for 30 minutes. One μl of the appropriate radiolabeled probe was then added and the samples (10 μl total volume) were incubated at room temperature for 30 minutes. Samples were run on a 6% polyacrylamide gel (pre-run for 30 minutes) at 80V for approximately 1.5 hours. The gel was dried and exposed to film.

### Chromatin immunoprecipitation assay

The chromatin immunoprecipitation (ChIP) assay was carried out as previously described [37] with the exception of the sonication step. The chromatin was sonicated in a dry ice/ethanol bath for 10 minutes at amplitude of 40% to generate DNA fragments between 150 and 350 bp in length. Samples included either 2 μg normal mouse IgG, 0.2 μl nuclear respiratory factor-1 (NRF-1) antibody [38] or 4 μg YY1 antibody (Santa Cruz sc-1703). PCR was performed using 5 μl of the ChIP-enriched DNA in a 25 μl reaction. The reaction was carried out for 34 cycles with an annealing temperature of 60°C. PCR products were stained with SYBR Green I nucleic acid gel stain (Invitrogen, Carlsbad, CA, USA) and run on a 2% low-melt agarose gel. The gel was visualized using a Typhoon scanner (GE Healthcare, Piscataway, NJ, USA) and the intensity of each band was quantified using ImageQuant software (Molecular Dynamics, Sunnyvale, CA, USA). Primer sequences are available on request.

### Cell invasion/migration assays

HS578T cells were split 1:6 and transfected the next day with 6 μg of either an empty vector (CMV-empty) or a YY1 overexpression vector (pCDNA3 HA-YY1) using TransIT-LT1 reagent (Mirus, Madison, WI, USA). After 72 hours the cells were treated with Versene-EDTA (Cambrex Bio Science, East Rutherford, NJ, USA) and were resuspended in migration buffer (DMEM without phenol red (Mediatech, Manassas, VA, USA), 1% BSA, 1 μM MgCl_2_, 0.2 μM MnCl_2_). Migration transwells (Corning, Lowell, MA, USA) and matrigel invasion chambers (BD Biosciences, Franklin Lakes, NJ, USA) were prepared according to the manufacturer's protocols. 10^5 ^cells were used for each migration assay and 5 × 10^4 ^cells for each invasion assay. Twenty-four hours later the migration and invasion chambers were cleaned with cotton-tip applicators and stained for 15 minutes in crystal violet solution (diluted 1:5 in dH_2_O from a stock of 2 mg/ml in methanol). The membranes were destained in dH_2_O and allowed to dry overnight. The stain was eluted from the migration membranes using 10% acetic acid, and the eluate was read at OD 600 nm in a Versamax microplate reader (Molecular Devices, Sunnyvale, CA, USA). The invasion membranes were removed, and mounted on slides using Permount (Fisher Scientific, Pittsburgh, PA, USA). The cells in 8 to 10 high-power fields (200× magnification) were counted and averaged for each membrane.

## Results

### Differential transcriptional activity at the HP1α gene promoter in MCF7 and HS578T cells

**Figure 1:**
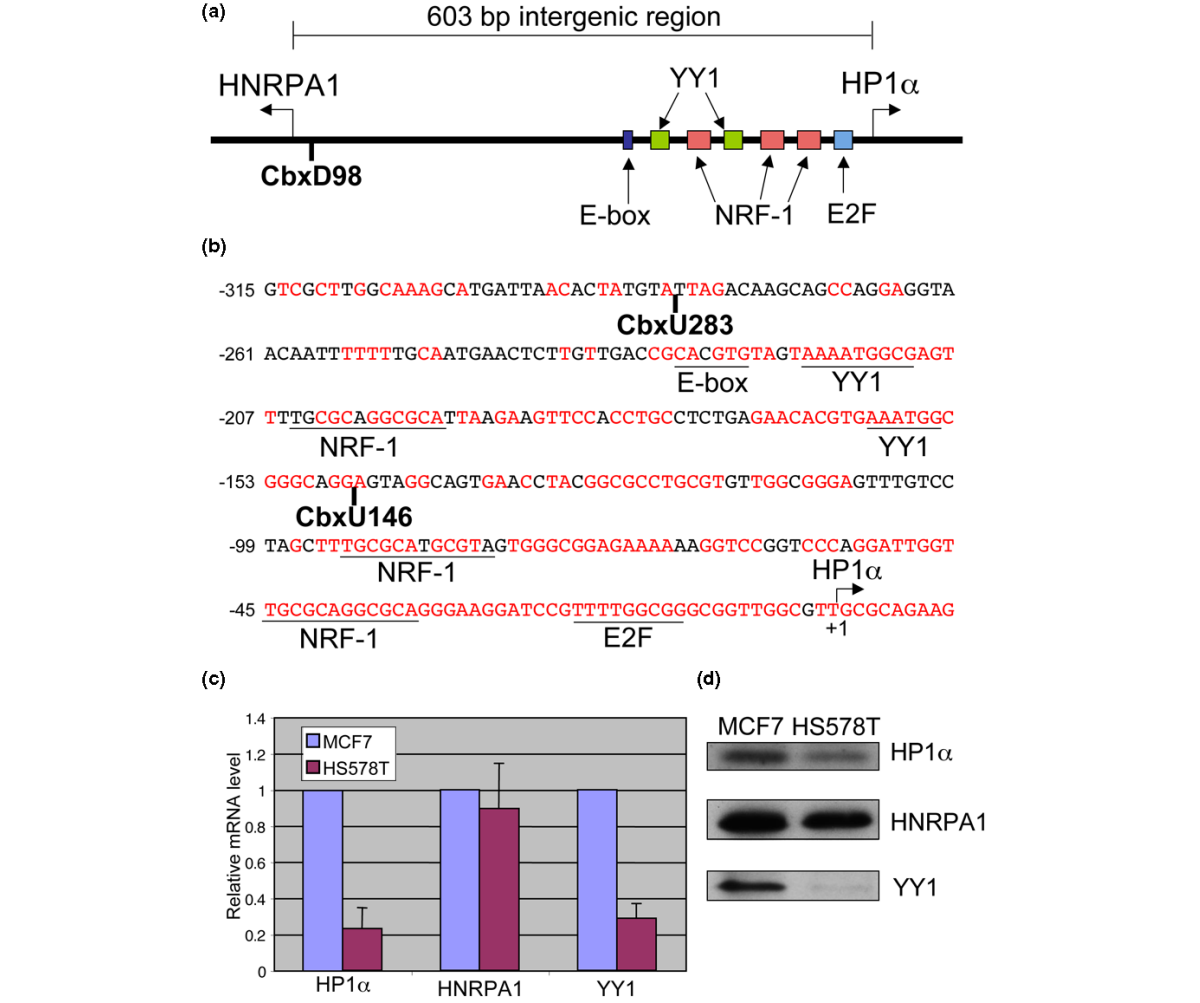
HP1α expression is down-regulated in the invasive cell line HS578T compared with the non-invasive cell line MCF7. **(a) **A schematic of the human HP1α gene promoter region. The positions of transcription factor binding motifs as well as the 5' end of the CbxD98 luciferase reporter construct are noted. **(b) **The sequence of the proximal HP1α gene promoter region. Nucleotides in red are conserved (identical) between human, mouse, rat and chimpanzee. Transcription factor binding motifs are underlined. The 5' ends of the CbxU283 and CbxU146 luciferase reporter constructs are noted. **(c) **mRNA expression of HP1α, heterogeneous nuclear ribonucleoprotein A1 (HNRPA1), and ying yang 1 (YY1) in MCF7 and HS578T cells. Quantitative RT-PCR results were normalized to the level of 28S ribosomal RNA. **(d) **Western blots showing protein levels of HP1α, HNRPA1, and YY1 in MCF7 and HS578T cells.

We cloned the upstream sequence of the human gene encoding HP1α, which shares a 603 bp intergenic region with the gene encoding heterogeneous nuclear ribonucleoprotein A1 (HNRPA1; Figure 1a). We used the UCSC genome browser [36] to look for areas of sequence conservation between species in the region between the HP1α and HNRPA1 gene transcriptional start sites. Part of the sequence of the intergenic region is depicted in Figure 1b. Nucleotides that are conserved between human, mouse, rat, and chimpanzee are indicated in red. Note the high degree of sequence conservation in the intergenic region. Next, we used the TESS database to identify potential transcription factor binding sites in this region (Figure 1b) [35]. The HP1α gene promoter has been shown to be a target of E2F1 and E2F4 as well as NRF-1 [37,39]. It is therefore not surprising that we identified a highly conserved E2F binding motif, as well as three highly conserved NRF-1 binding motifs. We also noted the presence of an E-box site, which has been suggested to be involved in differential expression of HP1α between MCF7 and MDA-MB-231 cells [40], and two highly conserved YY1 binding motifs. We performed quantitative RT-PCR analysis to determine relative mRNA levels of HP1α and HNRPA1 in MCF7 and HS578T cells. In agreement with previously published data [28,29], expression of HP1α mRNA and protein is lower in HS578T and MDA-MB-231 cells than in MCF7 cells (Figures 1c, d; data not shown). In contrast, we found that the expression of HNRPA1 mRNA and protein does not differ between these two cell lines (Figures 1c, d).

The relative difference in HP1α mRNA level between non-invasive and invasive breast cancer cell lines can be attributed to a difference in transcriptional activity or a difference in mRNA stability. To test if it is due to a difference in mRNA stability, we determined the mRNA half-life in MCF7 and MDA-MB-231 cells using actinomycin D treatment followed by quantitative RT-PCR. We found that the half-life of HP1α mRNA is approximately 12.5 hours in both cell lines (data not shown). Therefore, as a whole, our data suggest that there is a difference in transcriptional activity at the HP1α gene promoter between different cell lines.

### A YY1 site in the HP1α promoter region is involved in differential transcriptional activity between MCF7 and HS578T cells

**Figure 2:**
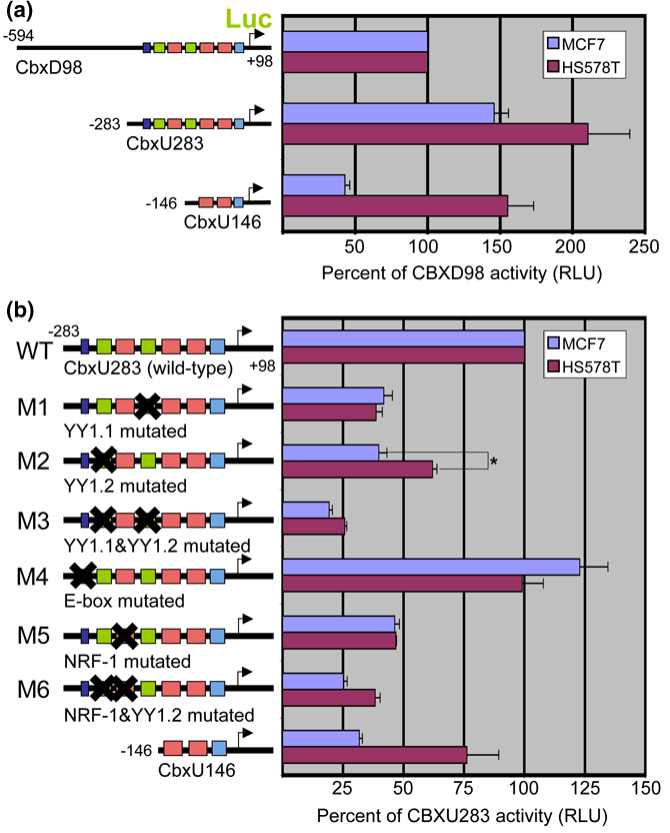
Mutation of a YY1 site in an HP1α gene promoter reporter construct has a more pronounced effect in MCF7 cells than in HS578T cells. **(a) **Reporter constructs representing the HP1α promoter region. Three HP1α constructs with the same 3' end and different truncations of the 5' end were transfected into MCF7 and HS578T cells. The luciferase activity of each construct is represented as a percentage of the activity of the CbxD98 construct. RLU = relative luciferase units. **(b) **Mutational analysis of the CbxU283 construct. The luciferase activity of each construct is represented as a percentage of the activity of the wild type construct. Only mutation of site ying yang 1 (YY1).2 consistently resulted in a differential effect in MCF7 and HS578T cells. *P *value was calculated using a two-sample unequal variance student t-test. * *P *= 0.002.

To determine the level of transcriptional activity at the HP1α gene promoter we sub-cloned the promoter region into a luciferase reporter construct. We made several luciferase constructs, one containing the entire bidirectional promoter region (CbxD98) and others containing truncations of the promoter at the 5' end (CbxU283 and CbxU146; Figures 1b and 2a). We also explored the possibility that sequence downstream of the start site may be involved in differential expression and found the sequence to have no effect on promoter activity (data not shown). Because it is difficult to directly compare luciferase activity levels between two cell lines, we decided instead to observe differences in the trend of luciferase activity of different constructs in each cell line. Therefore, the luciferase activity of each construct in Figure 2a is represented as a percentage of the activity of the CbxD98 construct. Truncating a portion of the promoter between -594 and -283 resulted in increased luciferase activity in both cell lines (compare CbxD98 and CbxU283 in Figure 2a). Truncation of the sequence between -283 and -146 caused a reduction in luciferase activity. Interestingly, this reduction in luciferase activity was more pronounced in MCF7 cells than in HS578T cells (compare CbxU283 and CbxU146 in Figure 2a). The results suggest that the region between -283 and -146 may be important for higher expression of HP1α in MCF7 cells as observed in Figure 1c.

To determine which binding motifs in the region between -283 and -146 may be important for differential promoter activity, we introduced mutations into four putative transcription factor binding sites in the region in the CbxU283 construct (Figure 1b). These binding sites included an NRF-1 site, two YY1 sites, and one E-box site. In Figure 2b, the luciferase activity of each mutant construct is displayed as a percentage of the activity of the wild-type CbxU283 construct. Each mutation had an effect on the promoter activity that was comparable in both cell lines; however, the only mutation that consistently had a differential effect between the two cell lines was the mutation in the YY1.2 site (the distal YY1 site, construct M2). The YY1.2 mutation caused a more pronounced reduction in promoter activity in MCF7 cells than in HS578T cells similar to what was observed for CbxU146 (Figure 2b). These results suggest that YY1 may play a role in the differential expression of HP1α between MCF7 and HS578T cells.

### YY1 is a positive regulator of HP1α expression

**Figure 3:**
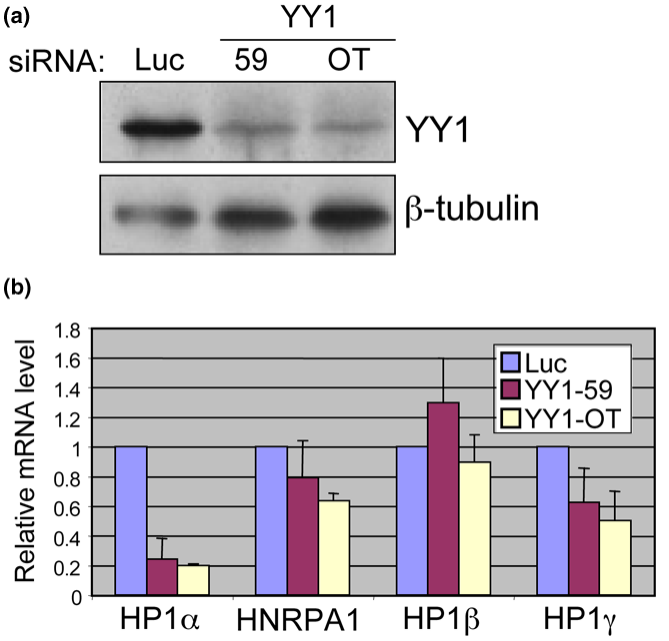
YY1 is a positive regulator of HP1α expression. **(a) **MCF7 cells were transfected with an siRNA targeting luciferase (Luc) or ying yang 1 (YY1; 59, OT), and protein and RNA were extracted five days later. Western blot shows levels of YY1 and β-tubulin following knockdown with the indicated siRNA. **(b) **Relative mRNA levels were determined using quantitative RT-PCR. Quantitative RT-PCR results were normalized to the level of 28S ribosomal RNA. HNRPA1 = heterogeneous nuclear ribonucleoprotein A1.

To determine what role YY1 plays in HP1α expression we performed siRNA knockdown experiments. MCF7 cells were transfected with siRNAs against YY1 or, as a control, an siRNA against luciferase. Two different YY1-specific siRNA oligos (59 and OT) were used to account for possible off-target effects. Five days after transfection with siRNA, protein and RNA were collected. Both of the YY1-specific siRNAs were effective in reducing the level of YY1 protein (Figure 3a). Quantitiative RT-PCR was used to determine the effect of YY1 knockdown on the expression of several mRNAs (Figure 3b). The level of HP1α mRNA was reduced significantly following YY1 knockdown. In contrast, the level of HNRPA1 mRNA was unaffected by YY1 knockdown. In addition, the levels of HP1β and HP1γ mRNAs were relatively unaffected by YY1 knockdown. The fact that YY1 knockdown had a specific negative effect on HP1α expression led to the conclusion that YY1 is a positive regulator of HP1α expression.

### Differential expression of YY1 leads to a lower level of YY1 DNA binding in HS578T cells compared with MCF7 cells

**Figure 4:**
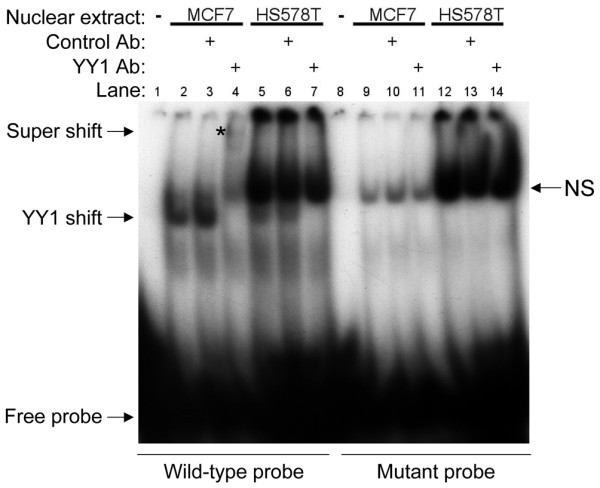
The YY1.2 mutation disrupts binding of YY1. Electrophoretic mobility shift assay was performed with a probe containing the ying yang 1 (YY1).2 sequence. Nuclear extract from MCF7 and HS578T cells (25 μg) was incubated with either a wild type probe or a mutant probe bearing the YY1.2 mutation. For super-shift experiments, control antibody (Ab; against Ac-H3K9) or an antibody to YY1 was added to the indicated sample. The super-shifted YY1-DNA complex is indicated with an asterisk. NS = non-specific.

The results of the luciferase experiments indicated that YY1 might play a role in the differential expression of HP1α in MCF7 and HS578T cells. An examination of the YY1 mRNA and protein levels showed that they are both reduced in HS578T cells compared with MCF7 cells (Figure 1c, d). This is significant, because our knockdown experiment indicated that YY1 is a positive regulator of HP1α expression. We used gel shift experiments to determine if a lower level of YY1 expression in HS578T cells resulted in decreased binding of YY1 to the YY1.2 site *in vitro*. Labeled probes containing the wild type or mutant YY1.2 sequence were incubated with nuclear extract from MCF7 or HS578T cells. The YY1-specific shift was only visible when we used MCF7 nuclear extract, indicating that there was not likely to be enough YY1 in the HS578T extract to produce a visible shift (Figure 4, compare lanes 2 and 5). The identity of the YY1-shifted species was confirmed by super-shift with an antibody against YY1 (Figure 4, lane 4 asterisk). Note the presence of a nonspecific band that was not shifted by the YY1-specific antibody. An unrelated antibody (against Ac H3K9) did not produce a super-shift (Figure 4, lane 3). The shifted band representing the YY1 protein-DNA complex was present only when the wild type probe was used, indicating that the mutation introduced into the YY1.2 site effectively disrupted YY1 binding (Figure 4, compare lanes 2 and 9). When more nuclear extract from HS578T cells was used, we observed a faint YY1 shift in the HS578T samples consistent with a low level of YY1 protein in these cells (data not shown).

### Differential occupancy of YY1 at the endogenous HP1α gene promoter region

**Figure 5:**
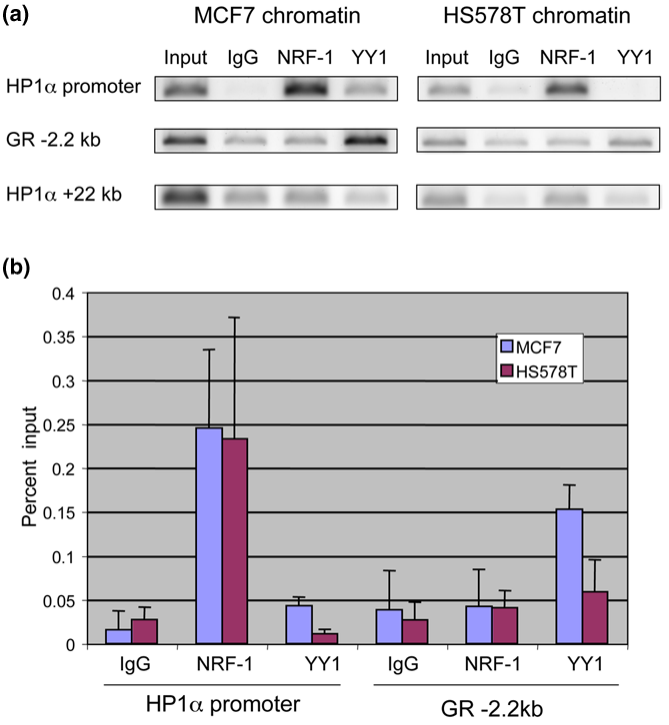
YY1 occupancy at the HP1α gene promoter is higher in MCF7 cells than in HS578T cells. **(a) **Chromatin immunoprecipitation (ChIP) assay was performed using chromatin form MCF7 and HS578T cells. Chromatin was immunoprecipitated using the antibodies indicated above each lane. PCR reactions were specific for the region indicated to the left of each panel. A region 2.2 kb upstream of the glucocorticoid receptor (GR) gene transcriptional start site was used as a positive control for ying yang 1 (YY1) binding. A region 22 kb downstream from the HP1α start site was used as a negative control. YY1 occupancy at the HP1α promoter can be observed only in the MCF7 samples. YY1 occupancy at the GR gene upstream regulatory region can be observed in both cell lines. **(b) **Quantification of ChIP data from at least three independent experiments using different chromatin preparations. NRF-1 = nuclear respiratory factor-1.

The gel shift experiment demonstrated that reduced expression of YY1 in HS578T cells results in reduced binding of YY1 to its DNA binding motif *in vitro*. Next, we wished to determine if this phenomenon also occurs at the endogenous HP1α gene promoter using the ChIP assay. Chromatin prepared from MCF7 and HS578T cells was immunoprecipitated using specific antibodies against YY1 or NRF-1. The HP1α gene promoter was previously identified as an NRF-1 target gene; thus, it served as a positive control [39]. NRF-1 occupancy was detected at HP1α gene promoter in both cell lines (Figure 5a). In contrast, YY1 occupancy at the HP1α gene promoter could only be detected in MCF7 cells. As a positive control for YY1 occupancy, we amplified a region in the glucocorticoid receptor gene promoter (located 2.2 kb upstream from the transcriptional start site), which contains three YY1 consensus binding motifs [41]. YY1 occupancy was detected at this region in both cell lines, indicating that YY1 DNA binding activity is not entirely absent in HS578T cells (Figure 5a). As a negative control, we amplified a region 22 kb downstream of the HP1α gene transcriptional start site. As expected, only background signal was detected in this region. We quantified the ChIP data from several experiments and calculated the occupancy of each transcription factor as a percentage of input (Figure 5b). From our ChIP experiments we conclude that the lower level of YY1 in HS578T cells results in reduced occupancy of YY1 at the HP1α gene promoter region. These studies indicate that the reduced occupancy of YY1 at the HP1α gene promoter in HS578T cells contributes to lower HP1α expression.

### YY1 overexpression reduces migration/invasion of HS578T cells *in vitro*

**Figure 6:**
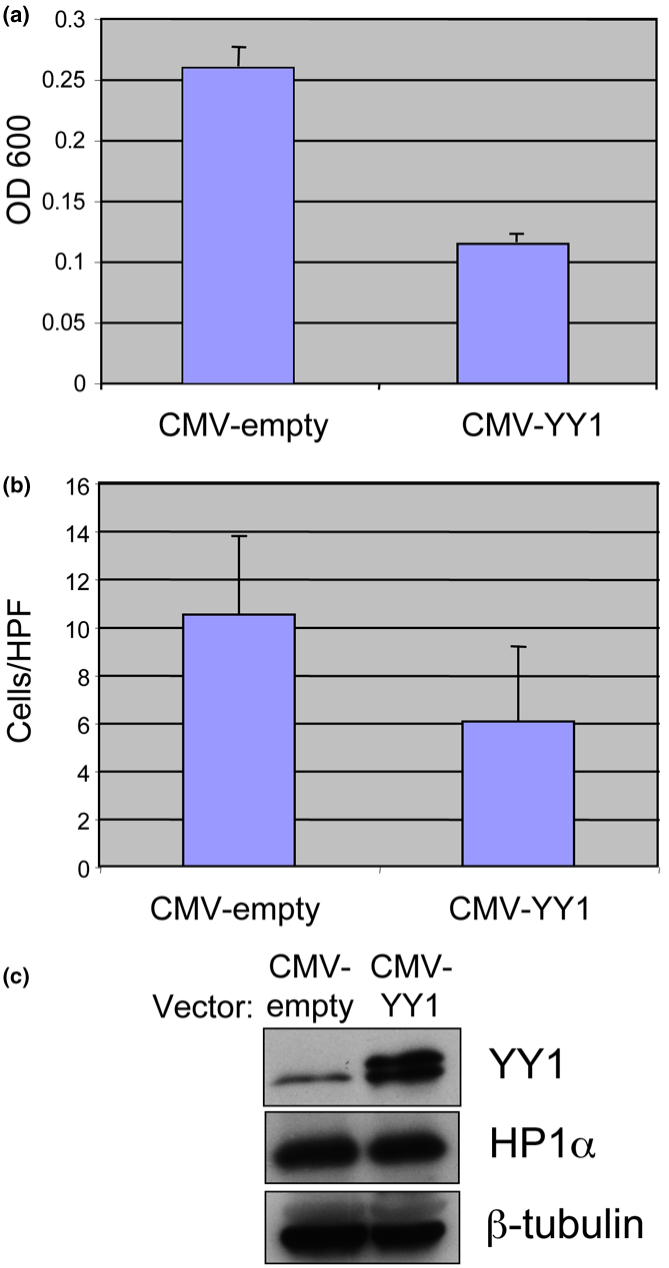
**YY1 overexpression suppresses HS578T cell migration *in vitro***. **(a) **HS578T cells were transiently transfected with either an empty vector (CMV-empty) or a vector expressing HA-tagged ying yang 1 (YY1; CMV-YY1). Seventy-two hours later the cells were used in migration assays using 5% fetal bovine serum as a chemoattractant. Membranes were stained with crystal violet and the stain was eluted with 10% acetic acid. The eluate was read at OD_600_. **(b) **The transfected HS578T cells were also used for *in vitro *matrigel invasion assays. Membranes were stained with crystal violet. Eight to ten high power fields (HPF; 200× magnification) were counted for each membrane and averaged. **(c) **An equal number of HS578T cells used for migration and invasion assays were boiled in SDS sample buffer, and an equal volume of each was run on an SDS-PAGE gel. Note that the upper band of the doublet in the YY1 panel represents the HA-tagged species, and the lower band represents endogenous YY1. No difference in HP1α expression was observed after 72 hours of YY1 overexpression. β-tubulin was included as a loading control.

HP1α has been identified as a suppressor of breast cancer cell invasion *in vitro *[29]. We have shown that YY1 is a positive regulator of HP1α expression. Next, we wanted to know if YY1 expression correlates with breast cancer cell invasiveness. The invasion of metastatic cancer cells *in vitro *requires two distinct cellular phenotypes: migration toward a chemoattractant and invasion through a matrigel matrix. These assays represent a simplified model of the metastatic process *in vivo*. We tested the effect of YY1 overexpression on both migration and invasion of the invasive breast cancer cell line HS578T. Cells were transiently transfected with either an empty vector or a YY1 expression construct and then subjected to *in vitro *migration and invasion assays. As shown in Figure 6, overexpression of YY1 caused about a 50% reduction in the migration of HS578T cells (Figure 6a). YY1 overexpression also led to a decrease in invasive potential of these cells (Figure 6b). The reduction in invasion was similar to the reduction in migration, indicating that the decreased invasion is due to the decreased migration. Interestingly, the decrease in migration/invasion occurred in the absence of detectable increase in HP1α expression (Figure 6c). We conclude that YY1 overexpression suppresses the migration of HS578T cells in a manner independent of HP1α expression.

## Discussion

The development and progression of cancer is due to changes in gene expression that result in the ability of cancer cells to proliferate autonomously, resist apoptosis, evade the immune system, and metastasize to distant sites [42]. In past decades, much work in the cancer field has focused on identifying genetic alterations that suppress or promote these phenotypes. It has become increasingly apparent, however, that disruption of proper epigenetic mechanisms also contributes significantly to cancer development [43]. For example, expression of HP1 in prostate cancer was found to be altered compared with normal prostate tissue [44]. It is therefore not surprising that HP1 has been linked to cancer progression in humans [45]. Although the molecular mechanism by which HP1α suppresses the invasive potential of breast cancer cells is unclear, it is important to understand the mechanism of reduced HP1α expression in invasive breast cancer cells.

In a previously published study, the DNA sequence of the HP1α gene in MCF7 and MDA-MB-231 was compared in order to identify any polymorphisms that may account for differential expression [40]. This study found no differences in the sequence in the HP1α gene between these two cell lines. In addition, no difference in the pattern of DNA methylation was found in the HP1α gene promoter region. The investigators therefore hypothesized, as we did, that the difference in HP1α expression is attributable to differences in transcription factor occupancy at the promoter region in different cell lines [40].

In the previous study, as in our study, luciferase reporter assays were used to determine how HP1α expression is differentially regulated at the level of transcription. One of the sequence motifs that was identified by this group was an E-box site (noted in Figure 1b). Mutation of this E-box site in the HP1α gene promoter construct resulted in a reduction in differential transcriptional activity between MCF7 and MDA-MB-231 cells [40]. In agreement with their data, we found that mutation of the same E-box site in our CbxU283 construct caused a small increase in promoter activity in MCF7 cells (Figure 2b, M4). However, we did not find that mutation of the E-box site had a differential effect on promoter activity of the CbxU283 construct between MCF7 cells and MDA-MB-231 cells (data not shown) or HS578T cells (Figure 2b).

Instead, we identified transcription factor binding motifs that were not identified in the previous study, including binding motifs for NRF-1 and YY1. Although our mutagenic analysis was not exhaustive, our data suggests that NRF-1 and YY1 are both important positive regulators in the expression of HP1α. Mutating both the distal NRF-1 and YY1 sites (NRF-1.3 and YY1.2) resulted in a drastic reduction in promoter activity (Figure 2b, M6). This result is supported by previously reported NRF-1 knockdown experiments [39], and by the YY1 knockdown data in this study (Figure 3b). YY1 has been implicated in the expression of many genes with numerous functions [46]. However, this is the first report to show that YY1 regulates HP1α expression. The role of YY1 as a positive regulator of HP1α expression implicates YY1 as an important gene in maintaining the cellular epigenetic program.

Our mutagenic analysis of the HP1α gene promoter region also revealed a possible role for YY1 in differential expression between MCF7 and HS578T cells. We found that the level of YY1 RNA and protein is higher in MCF7 cells than in HS578T cells (Figure 1c, d). Our ChIP data show that YY1 occupancy at the HP1α promoter region is much lower in HS578T cells than in MCF7 cells (Figure 5). Taken together, our data suggests that this difference in YY1 occupancy contributes to the difference in HP1α expression between the MCF7 and HS578T cell lines.

YY1 is a member of the GLI-Kruppel family of zinc-finger transcription factors [47]. YY1 is known to be essential for development because knockout of YY1 in mice results in peri-implantation lethality [48]. YY1 can act as an activator or repressor of transcription depending on its interaction with other factors [49,50]. Multiple studies have implicated YY1 in the development and progression of cancer [51,52]. Some of the most compelling data implicating YY1 in tumorigenesis are regarding its role in apoptosis. YY1 negatively regulates p53 by facilitating its interaction with Mdm2 (and its human orthologue, Hdm2), leading to p53 ubiquitination [53,54]. This mechanism is thought to increase the cell's resistance to apoptosis in response to genotoxic stress [54]. In addition, depletion of YY1 can sensitize cells to apoptotic stimuli through p53-independent pathways [46]. The fact that YY1 expression can render a cell resistant to apoptosis may explain why levels of YY1 are increased in certain types of cancer such as osteosarcoma, non-melanoma skin cancer, and acute myeloid leukemia [55-57].

Our data suggest that YY1 acts as a suppressor of migration in breast cancer cells. This is not the first report to implicate YY1 in invasion and metastasis. YY1 has been implicated in the metastatic progression of lung cancer cells due to its regulation of the putative metastasis suppressor HLJ1 [58]. YY1 has also been implicated in the negative regulation of the chemokine receptor CXCR4 [59], which has been implicated in the ability of breast cancer cells to metastasize to bone [60]. In addition, our results indicate that decreased YY1 expression can result in decreased expression of HP1α, which could contribute to the development of an invasive phenotype.

By definition, metastasis suppressor genes affect the metastatic process without affecting tumorigenesis [34]. As mentioned previously, YY1 has been proposed to play a number of roles in the process of tumorigenesis, and can therefore not be regarded as a pure metastasis suppressor. YY1 may play a role at several different points in cancer development and progression. High levels of YY1 may be advantageous to transformed cells during the early stages of cancer development, principally by reducing the tendency toward apoptosis. However, reduced expression of YY1 may be advantageous to metastasizing cells. Interestingly, high levels of YY1 are observed in high-grade prostate cancer, but prostate tumors with areas of low YY1 expression show a high rate of recurrence [61]. It is possible that decreased expression of YY1 allows sub-populations of cells within these tumors to become more highly invasive. By exploring the role of YY1 in migration and invasion, our study adds another layer to the complex role that YY1 may play in the development of metastatic disease.

## Conclusions

Studies have shown that the level of HP1α is reduced in invasive breast cancer cell lines such as MDA-MB-231 and HS578T compared with non-invasive cell lines. We found transcription factor YY1 to positively regulate HP1α gene expression through promoter analysis, siRNA-mediated knockdown and ChIP assays. Significantly, YY1 expression was detected to be lower in the invasive breast cancer cell line, implicating its role in the reduction of HP1α expression and possibly in the acquisition of an invasive phenotype.

## Abbreviations

BSA: bovine serum albumin; CD: chromodomain; ChIP: chromatin immunoprecipitation; CSD: chromoshadow domain; DMEM: Dulbecco's Modified Eagle Medium; HNRPA1: heterogeneous nuclear ribonucleoprotein A1; HP1: heterochromatin protein 1; NRF-1: nuclear respiratory factor-1; PBS: phosphate-buffered saline; RT-PCR: reverse transcription polymerase chain reaction; YY1: ying yang 1.

## Competing interests

The authors declare that they have no competing interests.

## Authors' contributions

JGL and NT designed the research. JGL, MK, and CNP performed the research. JGL, MK, CNP, and NT analyzed the data. JGL and NT wrote the paper.
